# Georgia Medical Association

**Published:** 1873-03

**Authors:** J. T. Johnson

**Affiliations:** Ass’t and Act. Secretary G. M. A.; Atlanta, Ga.


### GEORGIA MEDICAL ASSOCIATION.

ATLANTA, GA., March 19, 1873.

At the last meeting of the Georgia Medical Association, the following committees were appointed, and are expected to report at the ensuing meeting in Atlanta:

*On the Revision of the Constitution.*—Drs. A. W. Griggs, West Point; J. P. Logan, Atlanta; S. H. Stout, Atlanta; E. F. Knott, Griffin, and G. F. Cooper, Americus.

This committee will please refer to pages 14, 15, 21, and 22, of the Transactions of 1872.

*On Memorializing the Legislature with Reference to a Physician's Lien.*—First Congressional District, H. H. Smith, M.D., Screven county; Second Congressional District, W. L. Havis, M.D., Dougherty county; Third Congressional District, E. F. Colzey, M.D., Columbus; Fourth Congressional District, W. O'Daniel, M.D., Twiggs county; Fifth Congressional District, M. P. Dedwyler, M.D., Elbert county; Sixth Congressional District, R. M. Smith, M.D., Athens; Seventh, E. S. Ray, M.D., Atlanta.

(See page 7 of Transactions.)

*Committees of the Congressional Districts, appointed under the following resolution:*

"*Resolved, That the President of this Association appoint annually, from each Congressional District of this State, a Committee of three on the Practice of Medicine, a Committee of three on Surgery, and a Committee of three on Gynæcology, who shall report on some special subject or subjects, or make report of cases at their discretion, and present the same at the next meeting of this Association; and that individual members be requested to make voluntary individual reports in like manner.*"

### FIRST CONGRESSIONAL DISTRICT.

*Practice of Medicine.*—Dr. R. D. Arnold, Chatham county; Dr. J. J. Dearing, Chatham county; Dr. J. M. Cargil, Glynn county.

*Surgery.*—Dr. T. J. Charlton, Chatham county; Dr. C. Ganahl, Chatham county; Dr. R. J. Nunn, Chatham county.

*Gynæcology.*—Dr. J. S. Mimms, Screven county; Dr. J. B. Reed, Chatham county; Dr. H. H. Smith, Screven county.

### SECOND CONGRESSIONAL DISTRICT.

*Practice of Medicine.*—Dr. J. E. McMillan, Dougherty county; Dr. W. A. Strother, Mitchell county; Dr. P. L. Hillsman, Dougherty county.

*Surgery.*—Dr. B. M. Cromwell, Dougherty county; Dr. W. L. Davis, Dougherty county; Dr. Taliaferro Jones, Dougherty county.

*Gynæcology.*—Dr. G. F. Cooper, Sumter county; Dr. W. A. Green, Sumter county; Dr. Wm. Hardwick, Sumter county.

THIRD CONGRESSIONAL DISTRICT.

*Practice of Medicine.*—Dr. J. E. Bacon, Muscogee county; Dr. K. P. Moore, Crawford county; Dr. R. B. Ridley, Troup county.

*Surgery.*—Dr. W. H. Philpot, Talbot county; Dr. T. T. Smith, Houston county; Dr. M. W. Havis, Houston county.

*Gynæcology.*—Dr. V. H. Taliaferro, Muscogee county; Dr. F. A. Stanford, Muscogee county; Dr. T. C. Ellison, Talbot county.

FOURTH CONGRESSIONAL DISTRICT.

*Practice of Medicine.*—Dr. J. J. Caldwell, Pike county; Dr. A. H. Shi, Monroe county; Dr. C. H. Hall, Bibb county.

*Surgery.*—Dr. D. W. Hammond, Bibb county; Dr. B. F. Rudissill, Monroe county; Dr. C. S. Strother, Pike county.

*Gynæcology.*—Dr. J. T. Banks, Spalding county; Dr. W. F. Holt, Bibb county; Dr. J. R. Boon, Bibb county.

FIFTH CONGRESSIONAL DISTRICT.

*Practice of Medicine.*—Dr. H. H. Steiner, Richmond county; Dr. H. W. Hunter, Jefferson county; Dr. W. P. Dedwyler, Elbert county.

*Surgery.*—Dr. L. A. Dugas, Richmond county; Dr. A. G. Whitehead, Burke county; Dr. T. L. Lallerstedt, Richmond county.

*Gynæcology.*—Dr. W. H. Doughty, Richmond county; Dr. H. L. Battle, Jefferson county; Dr. S. C. Eve, Richmond county.

SIXTH CONGRESSIONAL DISTRICT.

*Practice of Medicine.*—Dr. R. D. Moore, Clarke county; Dr. B. F. Carleton, Greene county; Dr. W. V. Aderhold, Oglethorpe county.

*Surgery.*—Dr. H. H. Carlton, Clarke county; Dr. A. J. Shaffer, Gwinnett county; Dr. W. F. Howard, Clarke county.

*Gynæcology.*—Dr. R. M. Smith, Clarke county; Dr. T. S. Hutchinson, Oglethorpe county; Dr. C. W. Lang, Clarke county.

SEVENTH CONGRESSIONAL DISTRICT.

*Practice of Medicine.*—Dr. E. S. Ray, Fulton county; Dr. W. D. Hoyt, Floyd county; Dr. J. M. Johnson, Fulton county.

*Surgery.*—Dr. W. F. Westmoreland, Fulton county; Dr. Robert Battey, Floyd county; Dr. A. B. Calhoun, Coweta county.

*Gynæcology.*—Dr. J. P. Logan, Fulton county; Dr. D. G. Hunt, Gordon county; Dr. J. T. Jelks, Cobb county.

*Committee of Arrangements.*—Dr. E. S. Ray,* Dr. J. F. Alexander, Dr. W. G. Owen, Dr. E. L. Connally, Dr. J. M. Boring, Dr. C. A. Simpson, Dr. W. N. Judson.

*Committee on Publication.*—Dr. S. H. Stout, Secretary; Dr. J. T. Johnson, Assistant Secretary; Dr. W. C. Musgrove, Corresponding Secretary; Dr. W. O'Daniel, Treasurer; with an Advisory Committee of three appointed by the President, and consisting of Dr. J. P. Logan, Atlanta; Dr. E. S. Ray, Atlanta; Dr. J. B. Baird, Atlanta.

*Committee on Prize Essays.*—Dr. J. G. Westmoreland, Dr. J. P. Logan, Dr. W. H. Cumming, Dr. G. D. Sussdorf, Dr. W. R. Burgess.

*Committee on Education.*—Dr. J. G. Thomas, Dr. A. W. Griggs, Dr. F. A. Stanford, Dr. Robert Battey, Dr. J. F. Alexander.

*Committee on Inebriate Asylum.*—Dr. W. D. Hoyt, Dr. E. J. Kirkscey, Dr. G. F. Cooper.

*Committee on Medical Literature.*—Dr. H. F. Campbell, Dr. W. F. Westmoreland, Dr. W. H. B. Goodwin, Dr. R. D. Arnold.

*Committee on Climatology and Epidemic Diseases.*—Dr. W. A. Love, Dr. Carlisle Terry, Dr. William Duncan, Dr. A. L. C. Magruder, Dr. R. B. Ridley, Dr. W. A. Greene, Dr. A. B. Calhoun, Dr. R. D. Moore, Dr. J. M. Howell, Dr. DeSausseure Ford, Dr. Robert Battey, Dr. E. W. Alfriend.

*Committee on Medical Necrology.*—Dr. G. N. Holmes, Dr. R. P. Myers, Dr. G. F. Cooper, Dr. J. J. Mason, Dr. W. H. Hall, Dr. H. H. Carlton, Dr. J. R. McAfee.

(See page 21 of the Transactions of 1872.)

J. T. Johnson, M.D.,
*Ass't and Act. Secretary G. M. A.*

---

